# Longitudinal Multimodal Magnetic Resonance Imaging Reveals Improvement in Verbal Fluency Over Time in Moderate-to-Severe Traumatic Brain Injury

**DOI:** 10.1089/neur.2024.0149

**Published:** 2025-07-28

**Authors:** Ana Luiza Zaninotto, Fabiola Macruz, Fabricio S. Feltrin, Celi S. Andrade, Claudia C. Leite, Vinicius Monteiro de Paula Guirado, Wellingson S. Paiva, Sylvain Bouix

**Affiliations:** ^1^ Psychiatry and Neuroimaging Laboratory, Brigham and Women’s Hospital, Harvard Medical School, Boston, Massachusetts, USA.; ^2^ Department of Neurosurgery, Hospital das Clínicas da Faculdade de Medicina, Universidade de Sao Paulo (HC-FMUSP), Sao Paulo, Brazil.; ^3^ LIM44 Instituto de Radiologia (INRAD), Hospital das Clínicas da Faculdade de Medicina, Universidade de Sao Paulo (HC-FMUSP), Sao Paulo, Brazil.; ^4^ Neuroradiology Division, Department of Radiology and Oncology, Hospital das Clínicas da Faculdade de Medicina, Universidade de Sao Paulo (HC-FMUSP), Sao Paulo, Brazil.; ^5^ Rede D’Or São Luiz, Department of Radiology and Diagnostic, Sao Paulo, Brazil.; ^6^ University of Texas Southwestern Medical Center, Dallas, Texas, USA.; ^7^ Núcleo de Ensino, Pesquisa e Inovação da Alliar (NEPIA), Sao Paulo, Brazil.; ^8^ Software Engineering and IT Department, École de Technologie Supérieure, Montréal, Canada.

**Keywords:** cognition, diffuse axonal injury, language, magnetic resonance imaging, tractography, traumatic brain injury, verbal fluency

## Abstract

Most individuals with moderate-to-severe diffuse axonal injury (DAI) have impaired verbal fluency (VF) capacity. Still, the relationship between brain and VF recovery post-DAI has remained mostly unknown. The aim was to assess brain changes in 13 cortical thickness regions of interest (ROIs), fractional anisotropy (FA), and free water (FW) in three language-related tracts; the VF performance at 6 and 12 months after the DAI; and whether brain changes from 3 to 6 months predict VF performance from 6- to 12-month post-DAI. Twenty-one adults with moderate and severe DAI were analyzed. Structural and diffusion data were acquired on a 3T system 3 and 6 months after the injury. The differences in cortical thickness, FA, and FW values over time were analyzed as factors for the phonemic and semantic VF scores between the 6th and 12th months following the DAI. All analyses were corrected for multiple comparisons. Cortical thickness increased over time in 7 of the 13 ROIs in the right hemisphere and 5 of the 13 ROIs in the left hemisphere. There was an increase in FA in the right arcuate fasciculus and the inferior longitudinal fasciculus over time. An increase in phonemic VF scores was detected between 6 and 12 months post-traumatic brain injury, but not in semantic VF scores over time. Cortical thickness changes in the left posterior inferior frontal pars opercularis and left anterior superior temporal sulcus from 3 to 6 months were associated with improved phonemic VF scores over time. There was no association between diffusion magnetic resonance imaging metrics and VF scores. Our findings suggest that brain plasticity plays a significant role in the initial year following traumatic brain injury, as evidenced by increased cortical thickness and white matter integrity. Improved VF is associated with increased thickness in cortical motor regions responsible for speech performance. However, a larger sample size is needed to confirm these findings.

## Introduction

Diffuse axonal injury (DAI) is a primary pathology encountered in traumatic brain injury (TBI), mainly caused by the inertial acceleration/deceleration forces.^1,2^ Clinical characteristics following DAI are usually severe long-term cognitive and communication disability because of the widespread cortical and subcortical damage.^3–6^ One well-documented instrument to evaluate cognitive-communication deficits is the verbal fluency (VF) test.^7,8^ VF performance requires multiple cognitive processes, including verbal working and semantic memory, monitoring, and naming.^7,8^ Although cognitive decline is associated with brain atrophy, much evidence has suggested recovery of cognitive skills within the first few years.^9–15^ However, the brain mechanisms associated with this population’s cognitive regain are still debated.

Magnetic resonance imaging (MRI) plays a critical role in outlining the pathophysiological changes of the DAI over time.^16–21^ The characterization of cortical thickness is dependent upon the cortical region of interest (ROI),^22^ age,^23^ and the time from the brain injury.^24^ Participants with TBI and good clinical outcomes have shown an increase in cortical thickness in frontal areas, while a decrease in the cortical thickness has been evident in the TBI group with worse outcomes.^25,26^ Owing to the pathological characteristics of DAI, diffusion MRI (dMRI) is sensitive to detecting and monitoring microstructural brain changes.^27,28^ Fractional anisotropy (FA) is the most used dMRI-derived metric to estimate disruptions in fiber tracts.^29–32^ Free water (FW) uses a dual-compartment diffusion model approach that offers enhanced biological specificity compared to conventional diffusion tensor imaging.^33^ FW-corrected dMRI parameters, such as FW-corrected FA, have been demonstrated to have more specificity than standard non-FW-corrected FA due to their sensitivity to capture structural changes in the white matter that would be largely influenced by extracellular water and intravoxel crossing fibers.^34^ Changes in dMRI measures, including a reduction in FA and an increase in FW, have been reported in persons with neurodegenerative disorders and cognitive dysfunction.^14,34–37^

Despite the initial evidence of the dynamic changes in cortical and subcortical structures following brain damage, the long-term spontaneous neuroplasticity and its association with speech function and VF after a DAI remain poorly understood. In this study, we leverage a novel SpeechLabel cortical parcellation system to identify speech-related brain ROI,^38^ which includes a fine-grained subdivision of motor speech and sound perception cortex into 63 ROIs derived from the Directions Into Velocities of Articulators model.^39^ This cortical map has been previously used to support regional specificity of speech and communication decline in patients with neurodegenerative diseases.^40,41^

The current study intends to identify neuroimaging biomarkers that predict VF changes over time in persons with moderate-to-severe DAI due to a TBI. Our first aim was to evaluate changes in cortical thickness and dMRI measures (FA and FW) over time (at 3 and 6 months following TBI); the second aim was to determine changes in semantic and phonemic VF scores over time (at 6 and 12 months following TBI); the third aim was to verify if early changes (between 3 and 6 months post-injury) in cortical thickness and dMRI measures are associated with later changes in VF outcomes (between 6 and 12 months post-injury). Our prior study^42^ identified significant negative correlations at 6 months post-TBI between VF and dMRI metrics, including mean and radial diffusivity in the corpus callosum and axial and mean diffusivity in the superior longitudinal fasciculus. Notably, the current study employs methodological advancements compared to our initial work. This current study advances methodologically by: (i) utilizing FW-corrected dMRI metrics (e.g., FA), enhancing specificity by accounting for extracellular water and fiber crossing; (ii) separately analyzing phonemic and semantic VF, yielding nuanced insights into distinct language pathways; and (iii) focusing on language-related white matter tracts rather than whole-brain analysis. Therefore, leveraging this refined methodological approach in addition to the use of the SpeechLabel cortical parcellation Atlas,^38^ we hypothesize that the post-TBI brain changes in the initial months will serve as a predictive marker for a later VF recovery, improving clinicians’ ability to predict patients’ cognitive outcomes.

## Methods

This study is a hypothesis-driven retrospective analysis from a larger longitudinal study of cognitive changes in persons diagnosed with moderate and severe DAI. About 225 patients with TBI were admitted to the emergency room at the Hospital das Clinicas (HC-FMUSP), Brazil, from 2010 to 2015.^43^ The protocol was completed following the regulations and approved by the Institutional Ethics Committee, Comitê de Ética em Pesquisa CAPPESQ do Hospital das Clínicas da Faculdade de Medicina da Universidade de São Paulo (HC-FMUSP), number 0097/11; and by the Comitê de Ética em Pesquisa da Divisão de Psicologia do HC-FMUSP, number 18/2010. All participants gave written informed consent. The data selection for the current study was based on the quality of neuroimaging and the inclusion/exclusion parameters.

### Study design

MRI sessions were performed at 3 and 6 months following the brain injury. On the same day as the second MRI, the participants underwent the first cognitive assessment. The second cognitive assessment was performed 12 months after their injury. All the assessments were administered at the Institute of Radiology (INRAD HC-FMUSP).

### Participants

Participants with a possible or probable DAI diagnosis based on an acute computerized tomography scan at hospital admission were invited to return to the hospital for a 3-month MRI follow-up scan. Forty-three participants met the inclusion criteria and agreed to participate in the study. Four participants were excluded due to the presence of intra- or extra-axial hematomas or cerebral contusions larger than 10 mm at the initial computed tomography examination. Of the 39 remaining individuals who participated in the cognitive protocol, 18 were excluded due to loss of follow-up (*n* = 7), safety criteria for MRI examination (*n* = 4), dMRI artifacts (*n* = 5), deceased (*n* = 1), and epidural compressive hematoma (*n* = 1). The severity of the brain injury was based on their Glasgow Coma Scale scores and their post-traumatic amnesia (>24 h) at initial evaluation at the emergency room.

The participants were screened for MRI safety guidelines, and two certified neuroradiologists (F.F. and F.M.) confirmed the pathological characteristics of DAI prior to enrollment in this study. The severity of the DAI was characterized and classified according to the Marshall scale into grades III and IV by an expert radiologist (F.F.). We excluded participants with comorbid neurological diseases, such as stroke, previous TBI, previous psychiatric diagnosis, current drug abuse, contusions larger than 10 mm, midbrain shifts >5 mm, extra-axial fluid collection with compressive effect on brain structures, and contraindication for MRI.

### VF assessment

The VF scores were obtained at 6- and 12-month post-injury from the controlled oral word association test: *phonemic* and *semantic* VF tests.^8^ Participants were asked to provide as many words as possible within 1 min for each letter that began with F, A, or S (phonemic fluency test) or as part of a single category (semantic fluency test).

### Imaging data acquisition

Structural and diffusion data were acquired at 3- and 6-month post-injury on a 3T system (Intera Achieva, Philips Healthcare, Best, The Netherlands) using an 8-channel head coil (Philips Healthcare, Best, The Netherlands). The structural MRI consisted of a 3D T1-weighted Fast Field Echo (3DT1-FFE) with voxel resolution = 1 mm^3^. In addition, a set of two consecutive axial T1-weighted 3D gradient-echo pulse images was obtained (TR/TE = 3.6/7.3 ms, flip angle = 8◦, field-of-view [FOV] = 256 mm, acquisition matrix = 240 × 240; reconstruction matrix = 480 × 480; acquisition voxel = 1.0 × 1.0 × 3.0 mm; reconstruction voxel = 0.5 × 0.5 × 3.0 mm, 48 slices of 3.0 mm each, and acquisition time = 4 min). dMRI images were collected in the axial plane with gradients applied in 32 noncollinear directions (*b* = 1000 s/mm^2^). The entire brain was covered with 70 slices, 2 mm thick each, with no gaps in between. One image with no diffusion weighting was obtained (*b* = 0 s/mm^2^). Other parameters used were TR/TE = 8500/61 ms; matrix = 128 × 128; FOV = 256 × 256 mm; 2 mm^3^ isotropic voxel; NEX = 1; completion time = 7 min.

### Preprocessing

The raw T1-weighted data from the scanner were first converted from DICOM to NIFTI file format, followed by axis alignment and centering. Thorough visual inspection for completeness, distortion, and motion artifacts was performed using 3D Slicer (http://www.slicer.org; version 10).

### Cortical thickness

To extract reliable thickness estimates, T1-weighted images were processed with the longitudinal stream^44^ in FreeSurfer 7.1.0. An unbiased within-subject template space and image^45^ are created using robust, inverse-consistent registration.^46^ Several processing steps, such as skull stripping, Talairach transforms, atlas registration, spherical surface maps, and parcellations, are then initialized with standard information from the within-subject template, significantly increasing reliability and statistical power.^44^ Cortical thickness was determined as the closest distance between the gray matter and white matter boundary and the pial surface at each vertex of the cortical mantle.

**FIG. 1.**
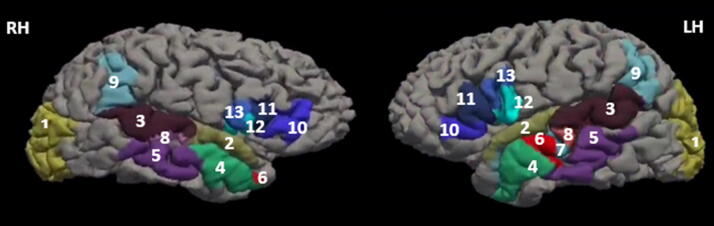
Selected regions of interest (ROIs) in both hemispheres derived from the SpeechLabel Parcellation Atlas. (1) occipital cortex; (2) anterior superior temporal gyrus (aSTg); (3) posterior superior temporal gyrus (pSTg); (4) anterior middle temporal gyrus (aMTg); (5) and posterior middle temporal gyrus (aMTg, pMTg); (6) anterior dorsal temporal sulcus (adSTs); (7) anterior ventral superior temporal sulcus (avSTs); (8) posterior dorsal superior temporal sulcus (pdSTs); (9) posterior supramarginal gyrus (pSMg); (10) anterior inferior frontal gyrus, pars triangularis (aIFt); (11) posterior inferior frontal gyrus, pars triangularis (pIFt); (12) ventral inferior frontal, pars opercularis (vIFo); and (13) dorsal inferior frontal, pars opercularis (pIFo).

The ROIs were derived from the SpeechLabel cortical atlas.^38,47^ Thirteen SpeechLabel parcellation ROIs for each hemisphere were selected for analysis: occipital cortex (OC); anterior and posterior superior temporal gyrus (aSTg, pSTg); anterior and posterior middle temporal gyrus (aMTg, pMTg); anterior dorsal, anterior ventral, and posterior dorsal superior temporal sulcus (adSTs, avSTs, pdSTs); posterior supramarginal gyrus (pSMg); anterior and posterior inferior frontal gyrus, pars triangularis (aIFt, pIFt); and ventral and dorsal inferior frontal, pars opercularis (vIFo, pIFo). The selected cortical ROIs, shown in Figure 1, have been chosen due to their connection to association tracts responsible for language networks (arcuate fasciculus [AF], inferior lateral fasciculus [ILF], and inferior occipitofrontal fasciculus [IOFF]), and by previous studies due to their likelihood of being affected in clinical speech impairment.^38,39,48^

### dMRI processing

After image reconstruction, a brain mask was created through a custom deep-learning tool specifically tuned for diffusion data.^49^ Images were corrected for motion and Eddy current distortion using FSL Eddy.^50^

Whole-brain tractography was performed using unscented Kalman filter tractography with a two-tensor FW model to account for crossing fibers and diffusion signals arising from FW.^51^ The resulting whole-brain tractography was then parcellated into 58 deep association and projection white matter tracts using fiber clustering.^52^

### Selected dMRI tracts

**FIG. 2.**
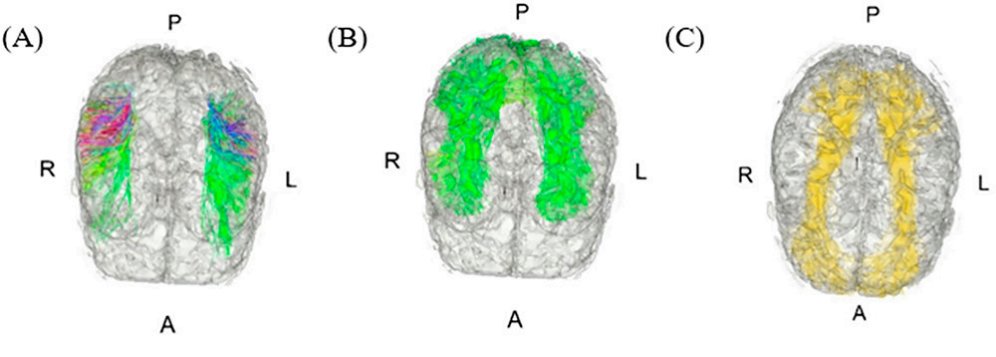
Representation of the white matter regions of interest: **(A)** arcuate fasciculus (AF), **(B)** inferior longitudinal fasciculus (ILF), and **(C)** inferior occipitofrontal fasciculus (IOFF). A, anterior; P, posterior; L, left hemisphere; R, right hemisphere.

Among the tracts provided by the parcellation framework of Zhang et al.,^52^ we selected three association tracts based on their role in language processing and VF, namely, the AF, ILF, and IOFF (Fig. 2). The AF represents the temporofrontal arching white matter pathway linking posterior and anterior language-related regions.^53^ Specifically, the AF projects from the superior temporal gyrus and Wernicke’s area (involved in speech comprehension) and curves around the Sylvian fissure to connect with Broca’s area (involved in speech production).^53^ The IOFF is part of the ventral pathway involved in speech and higher-level semantic representation, whereas the ILF has been associated with naming tasks^54,55^ and semantic working memory.^56^ The ILF is also part of a temporal lobe fiber network supporting language comprehension, together with the middle longitudinal fasciculus and extreme capsule as part of the ventral semantic stream.^57^

### Statistical analysis

To estimate the effect of time (3 and 6 months) on cortical thickness and dMRI-derived metrics (FA, FW) in the AF, ILF, and the IOFF and the effect of time (6 and 12 months) on phonemic and semantic VF, measured by the total number of words evoked in each task, we used a mixed-effect regression model.

To investigate if brain changes between 3 and 6 months after the injury predict VF changes from 6 to 12 months post-TBI. The brain measure changes over time (*delta values [Δ]*) were calculated as the difference between the cortical thickness ROIs measures and the Δ dMRI-derived metrics (FA and FW values) at 3 and 6 months following the brain injury.

 
Δ ROI cortical thickness=ROI cortical thickness at 6 months − ROI cortical thickness at 3 months

 
Δ dMRI-derived metrics=dMRI-derived metrics at 6 months − dMRI-derived metrics at 3 months

VF changes over time were calculated as the difference *(Δ)* between the results of VF (phonemic or semantic scores) at 12 and 6 months following the brain injury.

 
Δ semanticVF=semanticVF total score at 12 months − semanticVF total score at 6 months

 
Δ phonemicVF=phonemicVF total score at 12 months − phonemicVF total score at 6 months

The *Δ* values of the brain changes over time (between 3 and 6 months) were used to predict VF changes between 6 and 12 months following the brain injury. We used a mixed-effect model considering the difference in VF over time (*Δ* semanticVF and *Δ* phonemicVF) as the outcome and the changes in brain ROIs and dMRI-derived metrics over time (*Δ* ROI cortical thickness and *Δ* dMRI-derived metrics) as the predictor, adjusted by the baseline brain values.

All statistical models were adjusted by age and sex and each individual as a fixed factor, and *p* values were corrected for multiple comparisons using Bonferroni.

## Results

Twenty-one participants with moderate and severe DAI were adults between 18 and 48 years, 81% were male, with an estimated IQ median-low average. The demographic characteristics are displayed in Table 1.

**Table 1.** Demographic Characteristics of the Participants

Demographic variables	M ± SD	Range
Age	28.67 ± 10.1	[18–48]
Years of education	10.3 ± 3.1	[5–16]
Estimated IQ	86 ± 11	[79–112]
Sex (male), *n* (%)	17 (81%)	
Handedness (right), *n* (%)	18 (85.7%)	
Depression scores^a^	6.9 ± 6.3	[0–27]
Anxiety scores^b^	38.12 ± 7.3	[22–49]
Trauma severity (based on GCS score and PTA)^c^, *n* (%)		
Moderate	6 (28.6%)	
Severe	15 (71.4%)	
Trauma mechanism, *n* (%)		
Motorcycle accident	11 (52.4%)	
Automobile accident	6 (28.6%)	
Running over	3 (14.3%)	
Aggression	1 (4.8%)	

^a^ Back Depression Inventory (BDI).

^b^ State-Trace Anxiety Inventory–Trace version (STAI-T).

^c^ DAI severity based on the Glasgow Coma Scale score and post-traumatic amnesia at hospital admission.

DAI, diffuse axonal injury; GCS, Glasgow Coma Scale.

### Changes in cortical thickness ROIs over time

**FIG. 3.**
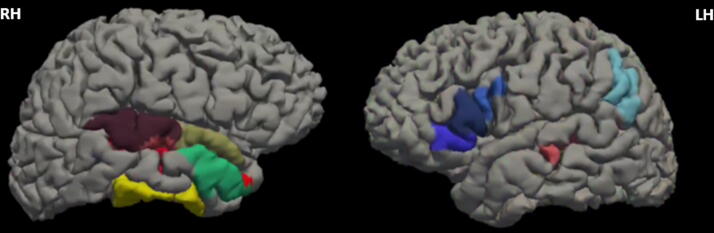
Cortical ROI showed a significant increase in cortical thickness from 3 to 6 months following the brain injury. ROI, region of interest.

As shown in Table 2, we observed a statistically significant cortical thickness increase between 3 and 6 months following the brain injury in seven ROIs from the right and five from the left hemisphere. The locations of the cortical ROIs increase are illustrated in Figure 3.

**Table 2.** Cortical Thickness Differences Between 3 and 6 Months Following DAI

ROI cortical thickness	RH								LH							
	M (SD) 3 months	M (SD)6 months	Coef.	SE	Z	[95% CI]	*p* ^*a*^	Adjusted *p*^*b*^	M (SD) 3 months	M (SD) 6 months	Coef.	SE	z	[95% CI]	*p* ^*a*^	Adjusted *p*^*b*^
Occipital cortex (OC)	2.05 (0.09)	2.07 (0.07)	0.020	0.013	1.56	[−0.005 to 0.046]	0.119	1.00	2.4 (0.12)	2.07 (0.11)	0.028	0.014	1.96	[0.000–0.056]	0.050	1.00
Anterior superior temporal gyrus (aSTg)	3.05 (0.33)	3.12 (0.31)	0.087	0.026	3.35	[0.036–0.138]	0.001	0.026	2.56 (1.11)	2.61 (1.13)	0.047	0.025	1.94	[−0.001 to 0.095]	0.052	1.00
Posterior superior temporal gyrus (pSTg)	2.86 (0.25)	2.90 (0.27)	0.077	0.022	3.55	[0.034–0.120]	<0.001	0.003	2.53 (0.59)	2.61 (0.62)	0.072	0.023	3.17	[0.027–0.116]	0.002	0.052
Anterior middle temporal gyrus (aMTg)	2.97 (0.25)	3.05 (0.30)	0.094	0.036	4.00	[0.048–0.140]	<0.001	0.003	2.45 (1.01)	2.55 (1.05)	0.091	0.029	3.14	[0.034–0.148]	0.002	0.052
Posterior middle temporal gyrus (pMTg)	2.77 (0.22)	2.840 (0.21)	0.090	0.022	4.07	[0.047–0.134]	<0.001	0.003	2.57 (0.22)	2.65 (0.25)	0.076	0.028	2.7	[0.021–0.132]	0.007	0.161
Anterior dorsal superior temporal sulcus (adSTs)	2.68 (0.33)	2.76 (0.28)	0.124	0.036	3.42	[0.053–0.195]	0.001	0.026	2.32 (0.99)	2.39 (1.03)	0.075	0.026	2.87	[0.023–0.127]	0.004	0.092
Posterior dorsal superior temporal sulcus (pdSTs)	2.60 (0.19)	2.65 (0.21)	0.070	0.020	3.39	[0.029–0.110]	0.001	0.026	2.38 (0.31)	2.45 (0.032)	0.077	0.021	3.63	[0.035–0.118]	<0.001	0.003
Anterior ventral superior temporal sulcus (avSTs)	2.55 (0.21)	2.61 (0.23)	0.102	0.024	4.23	[0.055–0.149]	<0.001	0.003	2.19 (0.93)	2.24 (0.96)	.058	0.032	1.82	[−0.004 to 0.120]	0.069	1.00
Posterior supramarginal gyrus (pSMg)	2.34 (0.16)	2.39 (0.15)	0.051	0.019	2.62	[0.013–0.090]	0.009	0.234	2.29 (0.13)	2.38 (0.13)	0.093	0.022	4.31	[0.051–0.135]	<0.001	0.003
Anterior inferior frontal, pars triangularis (aIFt)	2.39 (0.17)	2.44 (0.16)	0.070	0.030	2.35	[0.012–0.129]	0.019	0.494	2.41 (0.24)	2.57 (0.27)	0.159	0.034	4.67	[0.092–0.226]	<0.001	0.003
Posterior inferior frontal, pars triangularis (pIFt)	2.52 (0.28)	2.58 (0.22)	0.072	0.028	2.60	[0.018–0.126]	0.009	0.207	2.49 (0.19)	2.59 (0.18)	0.100	0.280	3.42	[0.41–0.150]	0.001	0.026
Dorsal inferior frontal, pars opercularis (dIFo)	2.44 (0.20)	2.49 (0.17)	0.071	0.029	2.49	[0.152–0.128]	0.013	0.299	2.42 (0.18)	2.51 (0.23)	0.086	0.023	3.69	[0.040–0.131]	<0.001	0.003
Ventral inferior frontal, pars opercularis (vIFo)	2.57 (0.22)	2.58 (0.23)	0.002	0.031	0.640	[−0.040 to 0.080]	0.521	1.00	2.47 (0.19)	2.56 (0.19)	0.087	0.032	2.7	[0.024–0.150]	0.007	0.182

^a^ Mixed-effect regression model adjusted for age and sex.

^b^ Family-wise error rate correction (FWER).

LH, left hemisphere; RH, right hemisphere; ROI, region of interest. *p*-value <0.05.

### Changes in dMRI-derived (FA and FW) metrics in white matter tracts over time

A significant increase of FA was observed in the right AF and the right ILF between 3 and 6 months post-injury. No differences were detected in FW values over time. The results are displayed in Table 3.

**Table 3.** Fractional Anisotropy and Free-Water Values in White Matter Tracts at 3- and 6-Month Post-DAI

		RH							LH							
	M (SD) 3 months	M (SD) 6 months	Coef.	SE	z	[95% CI]	*p* ^*a*^	Adjusted *p*^*b*^	M (SD) 3 months	M (SD) 6 months	Coef.	SE	z	[95% CI]	*p* ^*a*^	Adjusted *p*^*b*^
Arcuate fasciculus (AF)																
FA	0.575 (0.04)	0.578 (0.04)	0.018	0.004	4.11	[0.010 to 0.027]	<0.001	0.001	0.521 (0.04)	0.541 (0.04)	0.002	0.004	0.400	[−0.006 to 0.010]	.686	1.00
FW	0.118 (0.01)	0.115 (0.01)	0.0001	0.002	0.090	[−0.003 to 0.003]	0.926	1.00	0.112 (0.01)	0.112 (0.01)	−0.003	0.002	−1.64	[−0.006 to 0.0005]	.101	.301
Inferior longitudinal fasciculus (ILF)																
FA	0.466 (0.03)	0.473 (0.04)	0.010	0.004	2.41	[0.002 to 0.020]	0.016	0.048	0.442 (0.04)	0.452 (0.03)	0.007	0.007	1.08	[−0.006 to 0.020]	.279	.837
FW	0.146 (0.01)	0.142 (0.01)	0.003	0.002	1.51	[−0.001 to 0.007]	0.131	0.400	0.139 (0.01)	0.138 (0.01)	−0.005	−1.87	0.060	[−0.010 to 0.0002]	.061	.183
Inferior occipitofrontal fasciculus (IOFF)																
FA	0.578 (0.04)	0.586 (0.04)	0.001	0.006	0.000	[−0.012 to 0.012]	0.998	1.00	0.566 (0.05)	0.566 (0.05)	0.007	0.005	1.46	[−0.002 to 0.017]	.143	.430
FW	0.146 (0.01)	0.145 (0.02)	0.002	0.002	1.09	[−0.002 to 0.006]	0.926	0.830	0.146 (0.02)	0.146 (0.02)	−0.001	0.002	−0.330	[−0.005 to 0.004]	.742	1.00

^a^ Mixed-effect regression model adjusted for age and sex.

^b^ Family-wise error rate (FWER).

FA, fractional anisotropy; FW, free water; LH, left hemisphere; RH, right hemisphere. *p*-value <0.05.

### Changes in semantic and phonemic VF scores over time

**FIG. 4.**
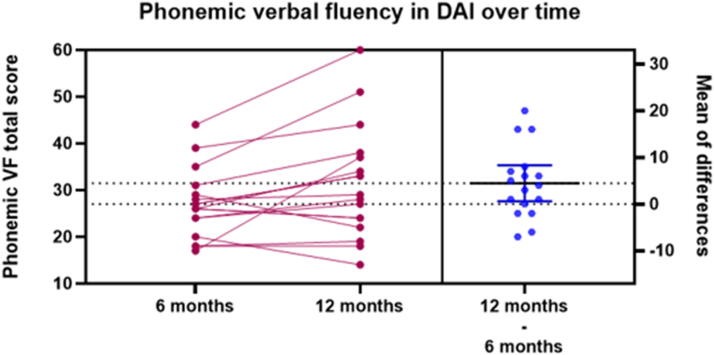
Phonemic VF total score improved from 6 to 12 months following the brain injury. VF, verbal fluency.

The participants’ average performance on the phonemic VF test was 27 ± 7.3 words at 6 months and 31 ± 11.8 words at 12 months, with a significant increase over time (β1 = 4.30, SE = 1.76, *z* = 2.44 [CI 95% 0.85, 7.75], *p* = 0.015, adjusted *p* = 0.03; Fig. 4). The average on the semantic VF test was 16.2 ± 3.0 words at 6 months and 16.6 ± 2.7 words at 12 months; however, no significant improvement was detected (β1 = 0.34, SE = 0.69, *z* = 0.49 [CI 95% −1.02, 1.70], *p* = 0.62, adjusted *p* = 1.00).

### Changes in cortical thickness and dMRI-derived metrics as predictors of VF improvement over time

**FIG. 5.**
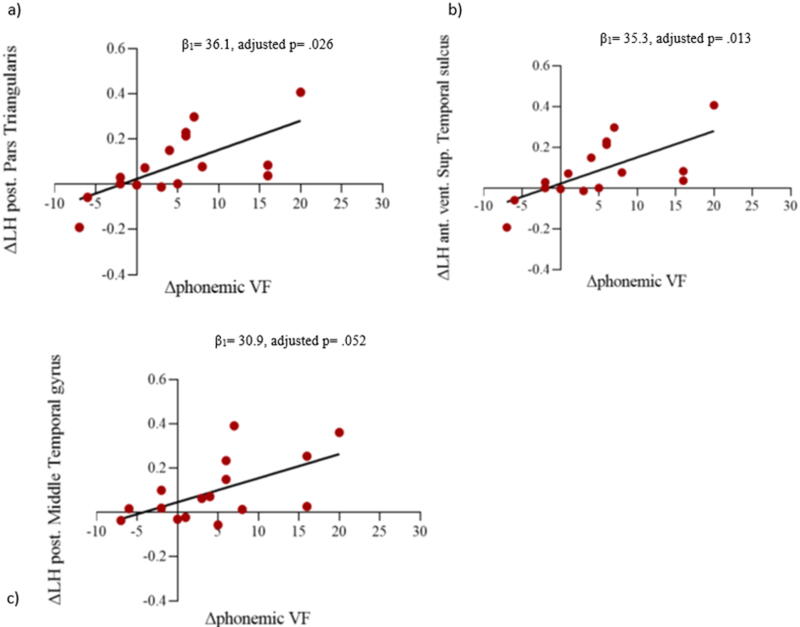
A positive association between the cortical thickness and Δphonemic VF scores: **(a)** Δ left posterior pars triangularis, **(b)** the Δ left anterior ventral superior temporal sulcus, and **(c)** Δ left posterior middle temporal gyrus.

Changes in cortical thickness between 3 and 6 months following the brain injury (Δ ROIs) in the left inferior frontal pars triangularis, in the anterior ventral superior temporal sulcus, and in the posterior middle temporal gyrus were positively associated with the changes in phonemic VF scores (between 6 and 12 months, Fig. 5). The complete results of the association between the changes in cortical thickness and the changes in VF tests over time are available as supplementary material (Supplementary Table S1a and b).

No significant association was detected between the changes in the white matter tracts measures and VF scores over time (Tables 4 and 5).

**Table 4.** Association Between Changes (Δ) in Fractional Anisotropy and Free Water (at 3- and 6-Month Post-Injury) and Δ in Semantic Verbal Fluency Scores (6- and 12-Month Post-Injury)

	RH						LH					
	Coef.	SE	z	[95% CI]	*p*	Adjusted *p*	Coef.	SE	z	[95% CI]	*p* *	Adjusted p**
Arcuate fasciculus (AF)												
FA	6.2	6.6	0.93	[−6.8 to 19.2]	0.35	1.00	3.3	6.1	0.54	[−8.7 to 15.3]	0.59	0.59
FW	114.75	147.7	0.78	[−174 to 404]	0.44	1.00	28.9	28.4	1.02	[−26.7 to 84.4]	0.31	0.93
Inferior longitudinal fasciculus (ILF)												
FA	4.96	7.4	0.67	[−9.5 to 19.4]	0.50	1.00	5.49	7.5	0.73	[−9.3 to 20.2]	0.47	0.56
FW	62.7	110.1	0.57	[−153 to 278]	0.57	0.85	24.6	23.5	1.05	[−21.3 to 70.6]	0.29	1.00
Inferior occipitofrontal fasciculus (IOFF)												
FA	2.33	6.22	0.37	[−9.9 to 14.5]	0.71	0.71	6.36	7.1	0.89	[−7.6 to 20.2]	0.37	0.75
FW	12.1	24.6	0.49	[−36.2 to 60.3]	0.62	0.75	18.0	22.3	0.81	[−25.8 to 61.7]	0.42	0.63

^*^ non-corrected *p*-value.

^**^ *p*-value corrected for multiple comparisons using Bonferroni.

FA, fractional anisotropy; FW, free water; LH, left hemisphere; RH, right hemisphere.

**Table 5.** Association Between Changes (Δ) in Fractional Anisotropy and Free Water (at 3- and 6-Month Post-Injury) and Δ in Phonemic Verbal Fluency Scores (6- and 12-Month Post-Injury)

	RH						LH					
	Coef.	SE	z	[95% CI]	*p* *	Adjusted p**	Coef.	SE	z	[95% CI]	*p* *	Adjusted p**
Arcuate fasciculus (AF)												
FA	6.46	14.2	0.46	[−21.3 to 34.3]	0.65	1.00	4.66	12.5	0.37	[−19.8 to 29.1]	0.71	0.71
FW	44.11	267	0.17	[−478 to 566]	0.87	0.87	29.63	58.5	0.51	[−85.1 to 144.3]	0.61	1.00
Inferior longitudinal fasciculus (ILF)												
FA	5.48	15.7	0.35	[−25.2 to 36.2]	0.73	1.00	11.53	15.1	0.76	[−18.1 to 41.1]	0.45	1.00
FW	−271.6	228	−1.19	[−719 to 176]	0.23	1.00	27.19	48.1	0.56	[−67.1 to 121.5]	0.57	1.00
Inferior occipitofrontal fasciculus (IOFF)												
FA	2.87	12.5	0.23	[−21.6 to 27.4]	0.82	0.98	5.86	14.6	0.40	[−22.7 to 34.5]	0.69	0.83
FW	−12.68	49	−0.26	[−108 to 83]	0.80	1.00	29.63	58.5	0.51	[−85 to 144]	0.61	1.00

^*^ non-corrected *p*-value.

^**^ *p*-value corrected for multiple comparisons using Bonferroni.

FA, fractional anisotropy; FW, free water; LH, left hemisphere; RH, right hemisphere.

## Discussion

Our results demonstrated a significant improvement in phonemic VF performance, but not in semantic VF scores over time. Bilateral cortical increase was observed in 7 of the 13 speech-related ROIs in the right temporal areas and 5 of the 13 ROIs increased in the left temporal and frontal areas between 3 and 6 months following the brain injury. The cortical changes in the left pIFt, avSTs, and pMTg were positively associated with phonemic VF score changes over time. An increase in FA in the right ILF and AF between 3 and 6 months post-injury was detected in our sample. No association was found between the changes in FA or FW values and phonemic or semantic VF scores over time.

Executive function, mostly related to prefrontal regions, is known to be a key component in decreased semantic and phonemic VF performance after a TBI^58,59^; however, additional cognitive components are involved in each subtype of VF tests. Semantic VF is interdependent on categorical/semantic networks and relies on long-term memory processes,^58^ semantic memory skills, and semantic networks.^60^ In fact, semantic VF tests are broadly used to support the diagnosis and monitor the disease progression in patients with mild cognitive impairment, Alzheimer’s disease, and aphasia.^59–61^ On the contrary, phonemic VF has been presumed to depend more on executive processes than semantic VF,^62,63^ including higher cognitive control, efficient organization of verbal retrieval and recall, self-monitoring, self-initiation, and inhibition of responses,^8,58^ making the phonemic VF test more sensitive to monitor cognitive changes after a brain injury than the semantic tests.

In this study, the increased cortical areas in the temporal and frontal regions may be attributed to cortical reorganization and regenerative mechanisms compensating for cognitive recovery.^64^ The right temporal region has been described to play a role in a functional and anatomical interface between lower-level auditory structures and higher-level association areas supporting abstract language aspects.^65^ The superior temporal gyrus and left inferior frontal gyrus play a key role in semantic memory retrieval, with an overlap between semantic and phonemic task demands.^66,67^ The increase in left cortical frontal areas observed in our study, including the left inferior frontal pars opercularis (IFo) and inferior frontal pars triangularis (IFt), usually referred to as Broca’s area, are key regions responsible for high-level control of orofacial and vocal motor function.^68^ The supramarginal gyrus has been related to the representation of internal and vocalized speech, with evidence for phonetic encoding.^69^ Although the temporal brain regions are usually associated with semantic performance,^70,71^ in our sample, just the left hemisphere changes over time were associated with phonemic performance. These results are in concordance with other studies that revealed an association between the left-brain areas (avSTs, pMTg, and IFt) and phonemic performance.^70,71^ Specifically, the left frontal areas are known to be the predominant site of language processing and articulatory networks^66,72^ with a prominent role in phonemic VF performance.^70,71^ The dMRI analysis revealed an increase in FA just in the right tracts. This asymmetry between increased FA among the brain hemispheres has also been previously reported, where the authors postulate a potential asymmetrical vulnerability of white matter tracts following a closed brain injury.^37,73^ Deviations in white matter tract symmetry levels indexed by changes in FA values are proposed as potential indicators of various neurological pathologies, such as multiple sclerosis,^74^ mild cognitive impairment,^75^ Alzheimer’s disease,^76^ brain tumors,^77^ and stroke.^78^ The decrease in FA in the right IOFF and ILF has been reported in veterans with at least one mild closed TBI.^73^ However, the authors also demonstrated a widespread decreased FA in the left AF, corticospinal tract, and bilateral IOFF as an effect of age.^73^ This previous report suggests that the right white matter tracts may be more vulnerable in closed TBI, which seems to explain some of our results. It is noteworthy that the age range of the participants in our study was between 18 and 48 years, which is not expected to have the confounding effect of aging on the FA values.

The AF, recognized as a major language pathway, interconnects key speech production processes and comprehension regions.^79^ In our study, the increase in FA in the right AF and ILF follows a consistent pattern with the increase in cortical thickness in the temporal regions. It appears to reflect neuronal plasticity and compensatory processes in interconnected brain areas.^22,26,28^ In animal models, behavioral and environmental exposure to post-brain trauma promotes neuroplastic mechanisms favoring brain recovery, including synaptogenesis, dendritic growth, gliogenesis, increased neuronal connections, and augmented glial and vascular cells^24,80^ and with a coherent arrangement of reactive astrocytes in rat models,^81^ which may reflect in the increase in the interconnected cortical regions.

The absence of significant changes in FW values over time in the AF, ILF, and IOFF observed in our sample does not preclude the possibility of further FW abnormalities in other brain regions or the late stages of the disease. Increased FW has been evidenced in TBI compared to controls.^36,82,83^ However, no effect of time on FW values has been identified in a TBI sample, with similar results accounting for TBI severity.^84^ Despite previous evidence suggesting that a widespread increase in FW in white regions predicted functional and cognitive outcomes,^36,85^ our results are concurrent with a longitudinal study that did not identify a significant association between FW values and neuropsychological performance at 2 weeks and 6 months after the brain injury.^84^ Our results also align with studies in stroke populations,^86,87^ reinforcing the notion that disruptions in white matter tracts may not necessarily correlate with phonemic VF performance.

Despite the study’s contributions, limitations are acknowledged. The small sample size diminishes statistical power. Still, the significant values after multiple comparison analyses correction may indicate robust findings. In addition, the restricted time points analyzed hinder establishing the initiation and cessation points of the observed brain reorganization pattern.

This study identified brain ROI changes as potential biomarkers that may serve as predictors of VF outcome. The examination of the temporal variability in specific brain ROIs could aid clinicians in predicting the course of a patient’s recovery. In our sample, we observed a natural improvement in VF; however, cognitive rehabilitation may be added as a supplementary intervention to further enhance cognitive recovery after a TBI.^88^ In conclusion, this study suggests a dynamic neuroplastic change following DAI, with an improved VF associated with increased left cortical thickness in oromotor regions. A larger sample size is needed to confirm these findings.

## Transparency, Rigor, and Reproducibility Statement

All imaging data were collected using standardized protocols and state-of-the-art neuroimaging techniques, ensuring high-quality and reproducible results. Furthermore, cognitive assessments were administered under controlled conditions, with validated measures to accurately capture cognitive functioning. To enhance transparency, we have provided detailed descriptions of our methodologies, including participant selection criteria, imaging protocols, and cognitive task assessments. Data processing and analysis were conducted following rigorous statistical standards to ensure the integrity of our findings. We are committed to promoting rigor and reproducibility in our research, and all relevant data and materials will be made available upon request to facilitate independent verification and further investigation by other researchers in the field.

## Abbreviations Used

AF: arcuate fasciculus
aIFt: anterior inferior frontal gyrus, pars triangularis
aMTg: anterior middle temporal gyrus
adSTs: anterior dorsal superior temporal sulcus
aSTg: anterior superior temporal gyrus
avSTs: anterior ventral superior temporal sulcus
BDI: Back Depression Inventory
CI: confidence interval
DAI: diffuse axonal injury
dIFt: dorsal inferior frontal, pars opercularis
dMRI: diffusion magnetic resonance imaging
FA: fractional anisotropy
FAt: Free Water corrected fractional anisotropy FW Free Water
FW: Free Water
FWER: Family-wise rate correction
GCS: Glasgow Coma Scale
IFo: inferior frontal pars opercularis
IFt: inferior frontal pars triangularis
ILF: inferior lateral fasciculus
IOFF: inferior occipitofrontal fasciculus
LH: left hemisphere
MRI: Magnetic resonance imaging
OC: occipital cortex
pIFt: posterior inferior frontal gyrus, pars triangularis
pMTg: posterior middle temporal gyrus
pSMg: posterior supramarginal sulcus
pSTg: posterior superior temporal gyrus
pdSTs: posterior dorsal superior temporal sulcus
PTA: Post-traumatic amnesia
RH: right hemisphere
ROI: region of interest
SE: standard error
STAI-T: State-Trace Anxiety Inventory-Trace
TBI: traumatic brain injury
VF: verbal fluency
vIFo: ventral inferior frontal, pars opercularis
