# Supplementary material for: Longitudinal Multimodal Magnetic Resonance Imaging Reveals Improvement in Verbal Fluency Over Time in Moderate-to-Severe Traumatic Brain Injury
Source: Neurotrauma Rep. 2025 Jul 28;6(1):600–12. doi: 10.1089/neur.2024.0149 (PMC12413258; doi:10.1089/neur.2024.0149)
Supplement: Supplementary Table S1 [file neur.2024.0149_supplementary_tables1.docx]

| Table 1a. The complete results with the association between the changes in cortical thickness (Δ ROIs) and the changes (Δ) in phonemic verbal fluency test | | | | | | | | | | | | | |
| --- | --- | --- | --- | --- | --- | --- | --- | --- | --- | --- | --- | --- | --- |
|  | RH | | | | | |  | LH | | | | | |
|  | Coef. | SE | Z | [95% IC] | p-value | adjusted p-value |  | Coef. | SE | Z | [95% IC] | p-value | adjusted p-value |
| Occipital cortex (OC) | 33.99 | 19.7 | 1.72 | [-4.65 72.6] | .085 | 1.00 |  | 26.6 | 18.72 | 1.42 | [-10 63.3] | .155 | 1.00 |
| Anterior superior temporal gyrus (aSTg) | 16.2 | 14.6 | 1.11 | [-12.4 44.8] | .267 | 1.00 |  | 25.9 | 11.1 | 2.33 | [4.1 47.6] | .020 | .260 |
| Posterior superior temporal gyrus (pSTg) | -2.5 | 17.2 | -.15 | [-36.3 31.3] | .885 | 1.00 |  | 31.3 | 13.3 | 2.35 | [5.2 57.4] | .019 | .247 |
| Anterior middle temporal gyrus (aMTg) | 5.8 | 13.3 | .44 | [-20.2 31.8] | .663 | 1.00 |  | 32.3 | 11.7 | 2.76 | [9.3 55.2] | .006 | .078 |
| Posterior middle temporal gyrus (pMTg) | -1.24 | 12.7 | - .10 | [-26.1 23.6] | .922 | 1.00 |  | 30.9 | 10.8 | 2.85 | [9.7 52.2] | .004 | **.052** |
| Anterior dorsal superior temporal sulcus (adSTs) | 9.12 | 14 | .65 | [-18.3 36.5] | .515 | 1.00 |  | 23.9 | 12.3 | 1.94 | [- .3 48] | .053 | .689 |
| Posterior dorsal superior temporal sulcus (pdSTs) | 13.8 | 13.3 | 1.03 | [-12.3 39.9] | .300 | 1.00 |  | 34.0 | 15.5 | 2.19 | [3.6 64.4] | .028 | .364 |
| Anterior ventral superior temporal sulcus (avSTs) | -2.87 | 12.6 | -.23 | [-27.5 21.8] | .820 | 1.00 |  | 35.3 | 9.8 | 3.59 | [16 54.5] | .0001 | **.0013** |
| Posterior supramarginal gyrus (pSMg) | 7.02 | 11.8 | .60 | [-16.1 30.1] | .551 | 1.00 |  | 13.9 | 14.4 | 0.96 | [-14.4 42.3] | .336 | 1.00 |
| Anterior inferior frontal, pars triangularis (aIFt) | -3.89 | 11.7 | -.33 | [-26.8 19.0] | .739 | 1.00 |  | 22.0 | 8.6 | 2.56 | [5.2 38.9] | .010 | .130 |
| Posterior inferior frontal, pars triangularis (pIFt) | 17.45 | 16.5 | 1.06 | [-15.0 49.9] | .291 | 1.00 |  | 36.1 | 11.5 | 3.13 | [13.5 58.7] | .002 | **.026** |
| Dorsal inferior frontal, pars opercularis (dIFo) | 5.87 | 16.7 | .35 | [-26.8 38.5] | .725 | 1.00 |  | 11.0 | 8.7 | 1.27 | [-6.1 28.1] | .206 | 1.00 |
| Ventral inferior frontal, pars opercularis (vIFo) | 21.3 | 16.5 | 1.29 | [-11.1 53.7] | .198 | 1.00 |  | .9 | 10.3 | 0.09 | [-19.7 21.6] | .931 | 1.00 |
|  |  |  |  |  |  |  |  |  |  |  |  |  |  |
| Table 1b. The complete results with the association between the changes in cortical thickness (Δ ROIs) and the changes (Δ) in the semantic verbal fluency test | | | | | | | | | | | | | |
|  |  |  |  |  |  |  |  |  |  |  |  |  |  |
|  | RH | | | | | |  | LH | | | | | |
|  | Coef. | SE | Z | [95% IC] | p-value | adjusted p-value |  | Coef. | SE | Z | [95% IC] | p-value | adjusted p-value |
| Occipital cortex (OC) | 0.91 | 8.3 | .11 | [-15.4 17.2] | .913 | 1.00 |  | 6.77 | 5.6 | 1.21 | [-4.2 17.8] | .227 | 1.00 |
| Anterior superior temporal gyrus (aSTg) | 6.36 | 5.8 | 1.1 | [-4.9 17.7] | .269 | 1.00 |  | 7.36 | 4.6 | 1.59 | [-1.7 16.4] | .111 | 1.00 |
| Posterior superior temporal gyrus (pSTg) | -10.5 | 6 | -1.75 | [-22.1 1.22] | .08 | 1.00 |  | 5.9 | 5.3 | 1.12 | [-4.4 16.2] | .263 | 1.00 |
| Anterior middle temporal gyrus (aMTg) | 2.69 | 4.85 | .55 | [-6.8 12.2] | .58 | 1.00 |  | 8.41 | 4.8 | 1.75 | [-1 17.8] | .080 | 1.00 |
| Posterior middle temporal gyrus (pMTg) | 2.13 | 4.9 | .43 | [-7.5 11.7] | .664 | 1.00 |  | 11.1 | 4.2 | 2.66 | [2.9 19.3] | .008 | .104 |
| Anterior dorsal superior temporal sulcus (adSTs) | .30 | 4.83 | .06 | [-9.2 9.8] | .950 | 1.00 |  | 9.1 | 4.5 | 2.02 | [.3 17.9] | .043 | .559 |
| Posterior dorsal superior temporal sulcus (pdSTs) | 2.53 | 5.28 | .48 | [-7.8 13.9] | .632 | 1.00 |  | 7.23 | 5.2 | 1.39 | [-3 17.4] | .165 | 1.00 |
| Anterior ventral superior temporal sulcus (avSTs) | -2.89 | 4.58 | -.63 | [-11.9 6.1] | .528 | 1.00 |  | 8.93 | 3.6 | 2.47 | [1.9 16] | .013 | .169 |
| Posterior supramarginal gyrus (pSMg) | 3.83 | 4.39 | .87 | [-4.8 12.4] | .384 | 1.00 |  | 4.06 | 5.2 | .79 | [-6.1 14.2] | .432 | 1.00 |
| Anterior inferior frontal, pars triangularis (aIFt) | 3.83 | 4.59 | .83 | [-5.16 12.8] | .400 | 1.00 |  | 6.92 | 3.24 | 2.13 | [.55 13.3] | .033 | .429 |
| Posterior inferior frontal, pars triangularis (pIFt) | 6.49 | 6.29 | 1.03 | [-5.8 18.8] | .300 | 1.00 |  | 11.3 | 4.7 | 2.39 | [2 20.5] | .017 | .221 |
| Dorsal inferior frontal, pars opercularis (dIFo) | 4.14 | 6.36 | .65 | [-8.3 16.6] | .514 | 1.00 |  | 2.38 | 3.7 | .65 | [-4.8 9.6] | .517 | 1.00 |
| Ventral inferior frontal, pars opercularis (vIFo) | 12.1 | 6.1 | 1.98 | [.15 24.1] | .047 | .611 |  | -2.99 | 4.2 | - .70 | [-11.2 5.2] | .474 | 1.00 |
|  |  |  |  |  |  |  |  |  |  |  |  |  |  |
